# Green Supply Chain Decisions Under Different Power Structures: Wholesale Price vs. Revenue Sharing Contract

**DOI:** 10.3390/ijerph17217737

**Published:** 2020-10-22

**Authors:** Xiaojing Liu, Wenyi Du, Yijie Sun

**Affiliations:** 1 Business School, Jiangsu Normal University, Xuzhou 221116, China; xjliu@jsnu.edu.cn (X.L.); xuzhousun1@163.com (Y.S.); 2 Management Science Institute, Hohai University, Nanjing 211100, China

**Keywords:** green supply chain, consumer preference, power structures, wholesale price contract, revenue-sharing contract

## Abstract

In the market, once consumers have a low-carbon preference, they will choose green low-carbon products. The market demand for green products is not only related to product price, but also consumers’ low-carbon preference. In this way, enterprise has to consider the cost of carbon emissions in the process of production and operation. In this paper, we consider a two-level supply chain system composed of a manufacturer and a retailer. The supply chain system can determine the price of products and the level of carbon emission reduction through different supply chain contracts: wholesale price contract and revenue sharing contract. However, the power control structure of a manufacturer and a retailer is different, which will further affect the decision-making strategy of the supply chain system. We set up four models (Wholesale Price—NM and NR, and Revenue-Sharing—SR and SM) of the supply chain with carbon emission reduction, and calculated and analyzed. The results show that firstly, regardless of whether the manufacturer’s power control structure or the retailer power structure is dominant, the manufacturer wholesale price with a contract on revenue-sharing is always higher than on wholesale price, and it is inversely proportional to the revenue-sharing proportion. Secondly, under the two power control structures, the carbon emission level of the manufacturer with a contract on revenue-sharing is always lower than on wholesale price, and it gradually decreases with the increase of the revenue-sharing proportion of the manufacturers. Thirdly, when the retailer dominates the supply chain, the retailer selling price with a contract on revenue-sharing is always higher than on wholesale price. Under the manufacturer’s power control structure, when the revenue-sharing ratio is small, the retailer selling price with a contract on revenue-sharing is higher than on wholesale price; when the revenue-sharing ratio is large, the retailer selling price with a contract on revenue-sharing is lower than on wholesale price. Finally, the validity of the model is verified by an example, and the sensitivity of the parameters is analyzed.

## 1. Introduction

In recent years, the carbon emissions produced by human beings in production and life have caused an imbalance in the earth’s ecology and caused global warming and other climate-related problems. The destruction of the ecological environment makes people pay more and more attention to environmental protection. Therefore, all countries are forced to pursue a low-carbon economy and sustainable development. Surveys have shown that consumers will more likely purchase products that contribute to environmental improvement. For example, a study by the UK Carbon Trust showed that consumers are more willing to buy low-carbon products, even if the price of low-carbon products is higher [1]. On the Alibaba platform, green consumers who focus on the ecological environment and can pay higher prices for green products had a compound growth rate of more than 80% from 2011 to 2015. These green consumers are willing to pay an average premium of 33% for green products [2]. These people can accept a little bit more and help reduce the industry’s carbon emissions in a more environmentally friendly way. Similarly, in Australia, Vanlay et al. studied the sales data of high-carbon and low-carbon products and found that the sales volume of low-carbon products was higher than that of high-carbon products [3]. Therefore, the improvement of consumers’ low-carbon awareness has a direct impact on the cost and operational decision-making of enterprise, thus posing a new challenge relating to supply chain operation management [4]. This means that the competitiveness of enterprise increasingly depend on whether they can meet the requirements of sustainable development [5]. For example, updating environmental protection equipment and investing in manufacturers for technical research and improvement. These methods increase manufacturing costs but can promote the market for environmentally friendly products. 

Additionally, the government can provide some guidance, such as policy guidance [6] and price subsidies [7,8], to promote the formation of a low-carbon economy and sustainable development. Outside the supply chain system, the government can provide some subsidies [9]. Another method is based on the internal perspective of the supply chain system. Because the product production process is realized through the form of a supply chain, the emission reduction decision of enterprise will be affected by the emission reduction decision of upstream and downstream enterprise [10,11]. To reduce the carbon emissions of products, it is necessary for all nodes of the supply chain to work closely together to find ways to reduce carbon emissions. Most manufacturing companies turn to partnerships with retailers [12,13]. The downstream retailer can expand sales and indirectly promote manufacturers to reduce emissions. This indirect way can allow for cooperation through revenue-sharing in the supply chain [14,15]. Sales incentives can increase the income of a manufacturer, so as to make up part of the cost. This also led to the company development, the stable development of the supply chain system and the sustainable development of a low-carbon society.

Low-carbon supply chain operation decisions are different from different supply chain contracts, for example, a revenue-sharing contract. Blockbuster video rental company first hired a revenue-sharing agreement, and achieved great success, thus attracting the attention of many scholars. This form of contract has been widely used in the supply chain [16,17]. In a dual-channel supply chain, revenue-sharing contracts are used to coordinate the conflicts of interest between different distribution channel providers [18]. In a closed-loop supply chain, revenue-sharing contracts are used to encourage product recycling [19]. In coordinating the cost and revenue competition between e-commerce companies and third-party logistics service providers, revenue-sharing agreements are also widely used [20,21]. In the field of the green supply chain, with which this study is concerned, Wang and Zhao designed a contract on revenue-sharing between a manufacturing company and retailer and realized a Pareto improvement based on consumers’ preference for low-carbon products and enterprises’ active implementation of measures to reduce emissions [22]. Cao et al. designed a contract on revenue-sharing based on different emission reduction policy environments to effectively improve the profits of supply chain members [23,24].

When faced with a powerful retailer, such as Walmart, Gome, a manufacturer, have no significant competitive advantage in the supply chain. The manufacturer has no initiative in terms of pricing, such as wholesale prices, in bargaining with the retailer [25,26]. The power control structure of a manufacturer and a retailer is different, which will further affect the decision-making strategy of the supply chain system. Therefore, under different power control structure, the paper studies how different supply chain contracts (wholesale price contract and revenue sharing contract) make green supply chain operation decisions, such as carbon emissions, wholesale prices, member performance, and so on.

**Figure 1:**
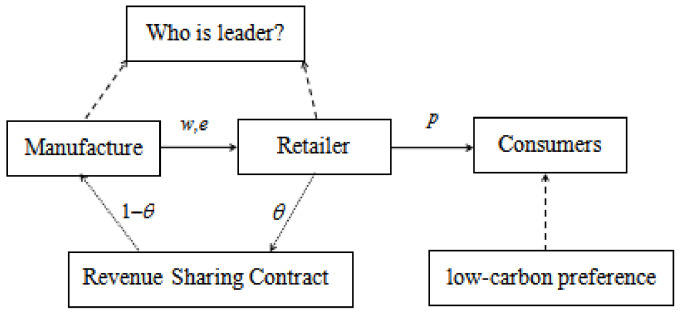
Production optimization in a low-carbon supply chain system.

In Figure 1, where w is the wholesale price of the unit product, *e* is the carbon-emission level of one unit of the product, *p* is the selling price of the unit product, θ is the proportion of revenue to the retailer.

Therefore, considering consumers’ preference for low-carbon products, this paper considers decision-making issues related to supply chain pricing and carbon emission reduction in various supply chain contracts. For a two-level supply chain system composed of a manufacturer and a retailer, we establish Stackelberg game models under different power control structures with different supply chain contracts. In the supply chain system, revenue-sharing contracts can be set or not set. If it is not set, it operates according to the traditional wholesale price contract. There are two main types of authorization in the supply chain: manufacturing supervisors and retailer supervisors. In this way, four supply chain structure models were formed gradually: The manufacturer power control structure with a contract on wholesale price (NM model), the retailer power control structure with a contract on wholesale price (NR model), the manufacturer power control structure with a contract on revenue-sharing (SM model), and the retailer power control structure with a contract on revenue-sharing (SR model). Additionally, we analyzed the solution and conducted strategies and determination of carbon emission reduction under different administrations with different revenue-sharing ratios, and how the different revenue-sharing ratio factors affect the best decision results and member performance.

Compared with the above research, the paper mainly innovates in the following three respects:

- What are the balanced emission reduction and pricing strategies for low trade prices and revenue-sharing contracts in a low-carbon supply chain?
- Which contract is better from a profit perspective? Which is the best way to reduce emissions?
- How does the distribution ratio affect the strategy and performance of revenue-sharing contracts?

The rest of this paper is organized as follows. In the second section, we provide a literature review. The third section is a description of the problem and some basic assumptions. In the fourth section, we establish four supply chain structure models and analyze the optimal solution in each case. In the fifth section, we analyze the size relationship of optimal decision strategies under wholesale price contracts and revenue sharing contracts. Through numerical analysis, the relationship between wholesale price, selling price, emission-reduction level, expected profit and parameter variables (sharing ratios) under the four modes are given in Section 6. In the seventh section, we provide the conclusion of the study and our contribution, and find the shortcomings of this paper and research directions in the future.

## 2. Literature Review

Each country is paying attention to its own carbon emission and making application contribution for the world green development. The production process of products involves carbon emission. Under different supply chain contracts, consumers’ low-carbon preference affects the decision-making of the supply chain, leading to different decisions of supply chain members. The literature review shows two related areas, including, supply chain decisions under consumers’ preference for low-carbon products and operation of a low-carbon supply chain with revenue-sharing.

### 2.1. Supply Chain Decision Under Consumers’ Low-Carbon Behavior

With the increased consumer demand for low-carbon products in the world, some scholars have studied supply chain decisions in this situation, and they have made some achievements. It shows that the majority of consumers can accept pay higher prices for low-carbon things [27,28]. Facing different environmental development in different countries, Letpath and Balakrishnan built two-game models to help enterprises improve the company marketing mix and increase profits [29]. Zhu et al. established a three-stage game model to discuss the influence of consumers’ environmental preferences on government subsidy policies and the green degree of enterprise products [30]. Combined with the environmental preferences of consumers, Liu et al. studied the decision-making behavior of manufacturers and retailers [31]. Benjaafar et al. analyzed how to decrease carbon emissions through the adjustment business activity decision problem and they studied the influence of enterprise cooperation on cost and carbon emission reduction in the supply chain [32]. 

Xiong et al. established a centralized and decentralized decision-making model, obtained the best combination of manufacturer and optimal retailer strategy under the rule of two decision-making issues, and discussed the influence of consumer environmental awareness on selling price [33]. Hammami et al. built an optimization model by incorporating carbon emissions into a Multi-level production inventory model limited delivery cycles [34]. Considering the impact of inventory policies, the total carbon emissions, the environment and other relevant parameters on demand, Hovelaque et al. proposed a model that can effectively reduce carbon emissions in production and operation [35]. Zhao et al. analyzed the Stackelberg game situation in which the manufacturer dominates suppliers and the situation of vertical cooperation between manufacturers and suppliers in the supply chain with the help of different games [36]. Based on the improved newsboy model, Liu et al. analyzed the influence of a government low-carbon emission reduction and price subsidy on the two-level supply chain [37]. 

By establishing a game system composed of government and suppliers, Cohen et al. discussed the consumer subsidy policy [38]. Under the centralized and decentralized structure, Li et al. found that the centralized structure is better than the decentralized structure for the supply chain and enterprise [39]. Wang et al. considered the government carbon emission tax policy in the supply chain system. They found that the centralized structure is superior to the decentralized structure of the supply chain and the company [40]. All of this is performed in a centralized and decentralized structure through the highest corporate and government decisions [41]. Ji et al. studied the emission reduction measures under the condition that consumers are interested in low-carbon products and sales channels [42]. The research shows that it is particularly feasible for the manufacturer and retailer to jointly reduce emissions. Hu et al. studied the impact of fair preference on the optimal pricing strategy in the situation of a retailer-dominated low-carbon supply chain [43].

### 2.2. Operation and Revenue-Sharing in a Low-Carbon Supply Chain

One way for the retailer and manufacturing company to cooperate is by entering into a revenue-sharing contract, which can help reduce emissions by increasing manufacturers’ market returns. Yang et al. studied the influence of revenue-sharing and first-time mobile advantages [44]. The results show that some researchers have compared the cost-sharing costs and contracts with or without CO2 tax, and the revenue-sharing contracts affect the supply chain performance [45,46]. Peng et al. studied the use of quantity control and transaction control for batch collaboration to achieve revenue-sharing and based on revenue-sharing to adjust and reduce emissions and design a subsidy mechanism [47]. Giovanni established a dynamic cooperation model of a closed-loop supply chain and studied the optimal green advertising input and pricing strategies of manufacturer and retailer under a contract on wholesale price and reverse contract on revenue-sharing [48]. Yang and Wang compared and analyzed the carbon emission reduction and earnings of the supply chain under decentralized, centralized and revenue-sharing contracts by building a two-level supply chain game model [49], which provided theoretical support for vertical cooperation and emission reduction on the upstream and downstream enterprise of the supply chain.

All of the above studies are based on the background of the dominant position of suppliers; some of them are related to operational decision-making issues in the green supply chain. These issues are under the guidance of retailers rather than under the same authorization. However, with some retailers, such as Wal Mart and Carrefour, entering the Chinese market, the market power of retailers has been expanding, and the supply chain mode dominated by the retailer has gradually emerged [50]. For example, in today’s society, there are not only power control structures dominated by manufacturers, such as Lenovo and Haier, but also power control structures dominated by a retailer, such as Suning and Gome. The research on supply chain management begins to consider the influence of the power differences between members in terms of supply chain decisions [51]. There was a real difference between the level of the advertising investment of the retailer and the level of the quality investment of manufacturers under different power control structures [52]. 

Additionally, it is found that the differences in power allocation influence on the channel profit of the supply chain recovery rate. The recovery rate is the highest when there is no leader in the market, and it is the lowest when the retailer is the leader. Thus, under different power control structures, is the incentive effect of choosing different contracts the same? What kind of impact will different revenue-sharing ratios have on pricing, carbon emission reduction and the revenue of green supply chain members under different power control structures? These questions will be the focus of our study.

## 3. Problem Description and Model Establishment

### 3.1. Problem Description

A two-level supply chain system consisting of a single manufacturer and a single retailer is considered. Among them, the manufacturer set wholesale prices and lowered emissions levels, and retailer sold goods to consumers at selling prices. Those who like low-carbon products. In addition to considering the price of products, they also pay attention to the carbon emission rate of products. In the environment of green economy, the price of products and consumers’ low-carbon preference will affect the change of product demand in the market. Therefore, on the one hand, this is exactly how consumers’ carbon behaviour is affected by the market demand for price-related, and on the other hand, by the level of carbon emissions level [53]. From the two types of research [54,55], the market demand is:

(1) $$D\left(p,e\right)=1-p+\gamma e$$

where *p* is the selling price one unit of the product, γ is the impact of the emission-reduction rate. At the same time, the research considers that consumers are more sensitive to prices than emission reduction levels. Therefore 0 < γ < 1,e is the carbon-emission level of one unit of the product.

The manufacturer needs to spend money to develop emission reduction technologies. In other words, the development of emission reduction technology requires a lot of investment. From the two types of research [56,57], the carbon-emission reduction cost is:

(2) Ce=ke22

where *k* is the effort coefficient of manufacturer carbon emission-reduction, to highlight the cost of reducing the emission-reduction, let k≥1.

### 3.2. Model Establishment

When the supply chain adopts a contract on wholesale price, the profits of the manufacturer and retailer are, respectively:

(3) $$\pi_{M}^{}=w\left(1-p+\gamma e\right)-\frac{1}{2}ke^{2}$$

(4) $$\pi_{R}^{}=\left(p-w\right)\left(1-p+\gamma e\right)$$

On the right side of (3), the first part is the wholesale income under the wholesale price contract, the second part is the cost of low-carbon production input. On the right side of (4) is the selling revenue of retailers under the wholesale price contract.

To encourage the manufacturer to operate with low carbon, the retailer can choose a contract on revenue-sharing to give some revenue subsidies to the manufacturer. Under the conditions of a contract on revenue-sharing, it can be assumed that the retailer will distribute a1−θ proportion of product revenue to the manufacturer and a θ proportion of revenue to itself after realizing the revenue. As the beneficiary of a low-carbon environment, the retailer may have enough motivation to make the manufacturer develop their carbon emission reduction level. They can transfer part of their income to the manufacturer to improve their profit level so that the manufacturer can more actively fulfill their carbon emission reduction responsibilities.

From the above assumption, when the supply chain adopts a contract on revenue-sharing, the profits of the manufacturer and retailer are, respectively:

(5) $$\pi_{M}^{\theta }=\left(w+\left(1-\theta \right)p\right)\left(1-p+\gamma e\right)-\frac{1}{2}ke^{2}$$

(6) $$\pi_{R}^{\theta }=\left(\theta p-w\right)\left(1-p+\gamma e\right)$$

On the right side of (5), the first part is the wholesale income under the revenue-sharing contract, the second part is the cost of low-carbon production input. On the right side of (6) is the selling revenue of retailers under the revenue-sharing contract.

## 4. Model Solution and Analysis

### 4.1. The Manufacturer Power Control Structure with a Contract on Wholesale Price (NM mode)

At this time, the manufacturer is the leader of the Stackelberg game and must first determine decision variables. Then the retailer determines the decision variables. We can use reverse induction to solve it. From the profit of the retailer (4), the second derivative of the wholesale price can be obtained: $\partial^{2}\pi_{R}^{}/\partial p^{nm}{}^{2}=-2<0$. It can be seen that the retailer profit is a concave function concerning the selling price, which can be obtained by its first-order condition:

(7) $$p^{nm}=\frac{1}{2}\left(1+\gamma e^{nm}+w^{nm}\right)$$

Next, the profit of the manufacturer can be obtained by substituting (7) for (3). At this point, the profit of the manufacturer is related to wholesale prices and carbon emissions. According to the properties of the Hessian Matrix, we can find that: $\partial^{2}\pi_{M}^{}/\partial w^{nm}{}^{2}=-1<0$. Then, $k-\gamma^{2}/4$ is greater than zero. In this way, the profit of the manufacturer is a combined concave function of the wholesale price and emission reduction level. Combined with the main terms, we can obtain the optimal wholesale price and carbon emissions, which are, respectively:

(8) $$w^{nm}{}^{*}=\frac{\gamma }{4k-\gamma^{2}}$$

(9) $$e^{nm}{}^{*}=\frac{2k}{4k-\gamma^{2}}$$

By substituting (8) and (9) for (7), the optimal wholesale price obtained:

(10) $$p^{nm}{}^{*}=\frac{4k-\gamma^{2}+2k\gamma +\gamma }{2\left(4k-\gamma^{2}\right)}$$

### 4.2. The Retailer Power Control Structure with a Contract on Wholesale Price (NR Mode)

As the leader of the Stackelberg game, the retailer determines its decision variables. Let *p* = w + t, where t is the price earning of one unit of the product, and let t > 0. The profit of the manufacturer can be reduced to:

(11) $$\pi_{M}=w^{nr}\left(1-w^{nr}-t+\gamma e^{nr}\right)-\frac{1}{2}ke^{nr}{}^{2}$$

Then, the second derivative of the profit of the manufacturer relating to the wholesale price and carbon emission level is solved. According to the properties of the Hessian matrix, when $w^{nr}=\gamma \left(1-t\right)/\left(2k-\gamma^{2}\right)$, the profit of the manufacturer is a combined concave function of the wholesale price and emission reduction level. At this time, according to the manufacturer first-order conditions concerning the wholesale price and emission reduction level, the optimal wholesale price and carbon emission reduction level can be obtained, which are, respectively:

(12) $$w^{nr}=\frac{\gamma \left(1-t\right)}{2k-\gamma^{2}}$$

(13) $$e^{nr}=\frac{k\left(1-t\right)}{2k-\gamma^{2}}$$

The expressions (12) and (13) are substituted for the profit of the retailer expression (3) and solved by reverse induction. the profit of the retailer is a concave function concerning t, which can be obtained by its first-order condition, so t=1/2. Then, t is substituted for the expressions of the wholesale price, carbon emission level and selling price, which are, respectively:

(14) $$w^{nr*}=\frac{\gamma }{2\left(2k-\gamma^{2}\right)}$$

(15) $$e^{nr*}=\frac{k}{2\left(2k-\gamma^{2}\right)}$$

(16) $$p^{nr*}=\frac{\gamma +2k-\gamma^{2}}{2\left(2k-\gamma^{2}\right)}$$

### 4.3. The Manufacturer Power Control Structure with a Contract on Revenue-Sharing (SM Mode) 

At this time, in the supply chain system, to encourage the upstream manufacturer to make innovations in carbon emission reduction technology innovation and share part of the revenue, the retailer can make up for the cost that the manufacturer incurs in reducing emissions due to technological innovation. As in Section 4.1, the manufacturer, as the leader of the Stackelberg game, determines decision variables. From the retailer profit, $\partial^{2}\pi_{R}^{\theta }/\partial p^{sm}{}^{2}=-2\theta <0$. the profit of the retailer is a concave function concerning the selling price, which can be obtained through the terms of the first order:

(17) $$p^{sm}=\frac{\theta +\theta \gamma e^{sm}+w^{sm}}{2\theta }$$

By substituting formula (17) for formula (5), the profit of the manufacturer can be obtained. the profit of the manufacturer is concerning the wholesale price and carbon emission reduction level. According to the properties of the Hessian matrix, we can find that:$\partial^{2}\pi_{M}^{}/\partial w^{sm}{}^{2}=-\left(1-\theta \right)/\theta <0$. Then, $w^{sm}{}^{*}=\gamma /\left(2\theta k-\gamma^{2}+2k\right)$, where the profit of the manufacturer is a combined concave function of the wholesale price and emission reduction level. Combining the first-order conditions, we can obtain the optimal wholesale price and carbon emission reduction level, which are, respectively:

(18) $$w^{sm}{}^{*}=\frac{\gamma }{2\theta k-\gamma^{2}+2k}$$

(19) $$e^{sm}{}^{*}=\frac{2k\theta^{2}}{2\theta k-\gamma^{2}+2k}$$

By substituting (18) and (19) for (17), the optimal wholesale price is

(20) $$p^{sm}{}^{*}=\frac{2k\theta^{2}-\theta \gamma^{2}+2\theta k+2\theta^{3}\gamma k+\gamma }{2\theta \left(2\theta k-\gamma^{2}+2k\right)}$$

### 4.4. The Retailer Power Control Structure with a Contract on Revenue-Sharing (SR Mode)

The same as 4.2, only when the supply chain system involves revenue-sharing contracts to make operational decisions. Let *p = w + T*, where T is the price earning of one unit of the product; and let T > 0. The profit of one manufacturer can be reduced to:

(21) $$\pi_{M}^{\theta }=\left[w^{sr}+\left(1-\theta \right)\left(w^{sr}+T\right)\right]\left(1-w^{sr}-T+\gamma e^{sr}\right)-\frac{1}{2}ke^{sr}{}^{2}$$

Then, the second derivative of the profit of the manufacturer concerning the wholesale price and carbon emission level is solved. According to the properties of the Hessian matrix, when $0<\gamma <\sqrt{2k/\left(2-\theta \right)}$, the profit of the manufacturer is a combined concave function of the wholesale price and emission reduction level. At this time, the solution of the optimal strategy is the same as Table 1. 

According to the manufacturer’s first-order conditions relating to the wholesale price and emission reduction level, the profit of the retailer is a concave function concerning *T*, which can be obtained by its first-order condition. Then, by substituting T for the expressions of the wholesale price, carbon emission level and selling price, the optimal pricing and carbon emission decision can be obtained. The specific results are shown in Table 2. 

And

$$\begin{array}{l} A_{1}=2\theta^{3}-6\theta^{2}+4\theta ;A_{2}=2\theta^{3}-8\theta^{2}+8\theta -1;A_{3}=\left(4k-2\right)\theta^{2}+\left(4-5k\right)\theta -1;A_{4}=4k\theta^{2}-6k\theta ;G_{1}=\theta^{2}-3\theta +2;\\ G_{2}=\theta^{2}-4\theta +4;\;G_{3}=\left(2k-1\right)\theta -3k+2;\;G_{4}=2k\theta -4k;\\ B_{1}=-2\theta^{4}+10\theta^{3}-17\theta^{2}+11\theta -2;\;B_{2}=-2\theta^{4}+\left(12+k\right)\theta^{3}-\left(3k+25\right)\theta^{2}+\left(2k+21\right)\theta -6;\\ B_{3}=\left(2-4k\right)\theta^{2}+\left(12k-8\right)\theta^{2}+\left(10-10k\right)\theta +3k;\;B_{4}=-4k\theta^{3}-\left(14k+2k^{2}\right)\theta^{2}-\left(14k+3k^{2}\right)\theta +5k;\;\\ C_{1}=-2\theta^{2}+5\theta -2;\;C_{2}=\left(k-2\right)\theta^{2}+\left(7-2k\right)\theta -5;\;C_{3}=2k\theta^{2}+\left(2-9k\right)\theta +4k;\;A_{4}=\left(2k^{2}-6k\right)\theta +6k. \end{array}$$

## 5. Sensitivity Analysis

**Proposition** **1.** *Under the wholesale price contract,* $w^{nm*}<w^{nr*.}$

**Proof of Proposition 1.**  According to the wholesale price in Equation (8) and (14), we can have: $w^{nm*}-w^{nr*}=-\frac{\gamma^{3}}{2\left(4k-\gamma^{2}\right)\left(2k-\gamma^{2}\right)}<0$, then $w^{nm*}<w^{nr*}$. □

Proposition 1 states that the wholesale price of the manufacturer under the retailer power control structure is higher than that under the manufacturer control structure. The main reason for this is that under the power control structure of the retailer, the manufacturer can adjust their strategies at any time according to the decision-making strategies of the retailer. The retailer will determine its specified selling price. As long as the manufacturer determines that the wholesale price is less than the retailer selling price, the retailer interests will coincide with those of the manufacturer. Therefore, at this time, the manufacturer will try to increase the wholesale price to be close to the selling price determined by the retailer.

**Proposition** **2.** *Under the wholesale price contract, when*$0<\gamma <2\sqrt{3k}/3$*, then*$e^{nm*}>e^{nr*}$*; when*$2\sqrt{3k}/3<\gamma <\sqrt{2k}$*, then*$e^{nm*}<e^{nr*}$.

**Proof of Proposition 2.**  According to the carbon-emission level in equation (9) and (15), we can have: $e^{nm*}-e^{nr*}=\frac{k\left(4k-3\gamma^{2}\right)}{2\left(4k-\gamma^{2}\right)\left(2k-\gamma^{2}\right)}$. When $0<\gamma <\frac{2}{3}\sqrt{3k}$, then $4k-3\gamma^{2}>0$,$e^{nm*}-e^{nr*}>0$; When $\frac{2}{3}\sqrt{3k}<\gamma <\sqrt{2k}$, then $4k-3\gamma^{2}<0$,$e^{nm*}-e^{nr*}<0$. □

Proposition 2 proved.

Proposition 2 indicates that the carbon emission of the manufacturer under the retailer power control structure is greater than that under the manufacturer control structure. The main reason is that under the power control structure of retailers, manufacturers can adjust their strategies at any time according to the decision-making strategies of retailers. At this time, consumers are more concerned about the price of products, and the carbon emission requirements of products are not too demanding. Because consumers understand that if the carbon emissions of manufacturers’ products are high, this will inevitably lead to higher wholesale prices, the retailer will correspondingly increase the selling price of products, which will finally be reflected in consumer preferences. Therefore, at this time, the manufacturer’s carbon emissions only depend on the manufacturer itself, or in other words, the retailer has no incentive effect on the manufacturer when they have a wholesale price contract.

**Proposition** **3.** *Under the wholesale price contract, when*$0<\gamma <\sqrt{2k-1}$*, then*$p^{nm*}>p^{nr*}$*; when*$\sqrt{2k-1}<\gamma <\sqrt{2k}$*, then*$p^{nm*}<p^{nr*}$.

**Proof of Proposition 3.**  According to the selling price in Equation (10) and (16), we can have: $p^{nm*}-p^{nr*}=\frac{\gamma k\left(2k-\gamma^{2}-1\right)}{\left(4k-\gamma^{2}\right)\left(2k-\gamma^{2}\right)}$. When $0<\gamma <\sqrt{2k-1}$, then $2k-\gamma^{2}-1>0$,$p^{nm*}-p^{nr*}>0$; When $\sqrt{2k-1}<\gamma <\sqrt{2k}$, then $2k-\gamma^{2}-1<0$, $p^{nm*}-p^{nr*}<0$. □

Proposition 3 is proved.

Proposition 3 shows the situation where a wholesale price sharing contract adopted in the supply chain system. When the carbon emission level has little impact on demand, the wholesale price of the retailer under the manufacturer’s power control structure is higher than that under the retailer power control structure. At this time, because the manufacturer’s rights are dominant, the manufacturer’s carbon emission level is high, and consumers with a preference for low-carbon products seldom choose to purchase high-carbon products, resulting in reduced demand in the market and higher prices. When the level of carbon emission has a great impact on, the wholesale price of the retailer under the manufacturer’s power control structure is smaller than that under the retailer power control structure. At this time, due to the dominant rights of the retailer, the retailer wants to encourage the manufacturer to reduce their carbon emissions, because low-carbon products attract more low-carbon preference consumers, resulting in increased demand in the market and reduced prices.

In this part, a sensitivity analysis is not provided because of the complexity of the solution strategy when the supply chain adopts a revenue-sharing contract. Then, in the sixth part, in the context of a market environment, some assumptions are made to provide the operation strategy and profit change of a low-carbon supply chain when a revenue-sharing contract is adopted.

## 6. Numerical Analysis and Discussion 

We conducted a more detailed inspection and analysis of the above methods and theorems and tried our best to make the calculation process easier and more convenient in dollars. It is assumed that the manufacturer’s carbon-emission-reduction cost coefficient is k = 1, and the impact of the carbon emission rate has a small (γ=0.3) and large (γ=0.7) impact on the demand. After calculation, Table 3, Table 4, Table 5, Table 6 and Table 7 shows the impact of the change in revenue share on the decision results and the profits of supply chain members.

Table 3 shows that there are two consequences of the level of carbon emissions having little impact on demand. Firstly, whether it is under the power control structure of the manufacturer or that of the retailer, the wholesale price of the manufacturer with revenue-sharing contracts is always higher than that of the manufacturer with a contract on wholesale price. Secondly, under a revenue-sharing contract, when the manufacturer’s power is dominant, the manufacturer wholesale price is a decreasing function of the revenue-sharing proportion. The larger the retailer revenue-sharing with the manufacturer, the lower the manufacturer price will be for the retailer. On the contrary, when the retailer power is dominant, the wholesale price of the manufacturer will first increase, and then decrease with the increase of the revenue-sharing ratio. The overall trend is a decrease. If the revenue-sharing ratios are either too small or too large, as in the case under the retailer power control structure, this will cause the manufacturer to have a lower wholesale price for the retailer.

There are two consequences of the level of carbon emissions having a great impact on demand. Firstly, whether it is under the power control structure of the manufacturer or that of the retailer, the wholesale price of the manufacturer with a contract on revenue-sharing is always higher than that on wholesale price. Secondly, under the revenue-sharing contract, when the manufacturer’s power is dominant, the wholesale price of the manufacturer is a decreasing function of the revenue-sharing proportions. The larger the revenue-sharing of the retailer with the manufacturer, the lower the manufacturer price will be for the retailer. When the retailer power is dominant, the wholesale price of the manufacturer is a decreasing function of the revenue-sharing proportions. The larger the revenue-sharing of the retailer with the manufacturer, the lower the manufacturer wholesale price will be for the retailer.

On the whole, regardless of the impact of the carbon emission level on demand, when a revenue-sharing contract is adopted, under the same revenue-sharing proportion, the wholesale price of the manufacturer in the retailer power mode is higher than that in the manufacturer power mode. Their size relationship is SR > SM > NR > NM.

Table 4 indicates that there are two consequences, irrespective of how the carbon emission level affects demand. Firstly, whether it is under the manufacturer’s power control structure or the retailer power control structure, the carbon emission level of the manufacturer under the revenue-sharing contract is always lower than that under the wholesale price contract. Secondly, under the revenue-sharing contract, whether the manufacturer’s power is dominant, or the retailer power is dominant, the manufacturer’s carbon emission level is a decreasing function of the revenue-sharing proportion. The less the retailer shares with the manufacturer, the greater the manufacturer’s carbon emission level.

When the supply chain adopts a revenue-sharing contract, under the same revenue-sharing proportion, the manufacturer’s carbon emission level in the retailer power mode is lower than that under the manufacturer’s power mode. This shows that revenue-sharing is conducive to manufacturers reducing their carbon emissions and plays an incentive role. When the carbon emission level has little influence on the demand, their size relationship is NM> NR > SM > SR. When the carbon emission level has a great impact on the demand, the carbon emission level is NM > NR > SM > SR under a lower sharing ratio. With the increase of the sharing ratio, the size relationship changes into NM> NR > SR > SM. When the sharing ratio increases by a large amount, the carbon emission level is NM> SM > SR > NR. Under the revenue sharing contract, we find that the carbon emission level is increased, which has nothing to do with the power structure.

Table 5 shows that when the revenue-sharing ratio is small, the retailer selling price is always higher than that of the wholesale price contract, either under the manufacturer’s power control structure or the retailer power control structure. When the proportion of revenue-sharing is large, under the manufacturer’s power control structure, the retailer selling price with a contract on revenue-sharing is lower than that on wholesale price. The retailer selling price with a contract on revenue-sharing is higher than that on wholesale price. If a revenue-sharing contract is not implemented, the retailer is willing to allow the manufacturer’s dominant power, which is more beneficial for its selling price, at this time, NM > NR. If a revenue-sharing contract is implemented, when the revenue-sharing ratio is very small or large, the retailer is still willing to allow the manufacturer power dominant, which is more beneficial for its selling price, at this time, SM > SR. When the revenue-sharing ratio is moderate, the retailer is willing to make its power dominant, which is more beneficial for its selling price, and at this time, SR > SM.

Table 6 shows that when the carbon emission level affects demand negatively, the profit of the manufacturer with a contract on revenue-sharing is always greater than that on wholesale price, regardless of whose power is dominant. It can be understood that consumers are not very sensitive to low-carbon products. A manufacturer can reduce carbon emissions or not.

When the proportion of revenue-sharing is smaller, the expected profit of the manufacturer with a contract on revenue-sharing is less than that with a contract on wholesale price. When the proportion of revenue-sharing is large, the expected profit of the manufacturer with a contract on revenue-sharing is greater than that with a contract on wholesale price. Under the power control structure model of the retailer, when the proportion of revenue-sharing is large or small, the expected profit of the manufacturer with a contract on revenue-sharing is smaller than that with a contract on wholesale price. When the proportion of revenue-sharing is moderate, the expected profit of the manufacturer with a contract on revenue-sharing is larger than that with a contract on wholesale price. It can be understood that for revenue-sharing contracts, the manufacturer will adjust its strategies at any time to deal with different revenue-sharing ratios of the retailer, and the two sides are also looking for a revenue-sharing ratio coefficient in the process of gradual cooperation. On the whole, when there is no revenue-sharing contract, the cost of carbon emission technology accounts for a part of the profit, which makes the profit level low and will gradually diminish the enthusiasm for low-carbon innovation. Once the revenue-sharing incentive measures are implemented, the profit of the manufacturer will be improved more quickly.

Table 7 shows that the more consumers prefer low-carbon products, the greater their expectations under the same sharing ratio. There are two consequences of the level of carbon emissions having little impact on demand. Firstly, regardless of whether the manufacturer’s power control structure or the retailer power is dominant, the profit of the retailer with a contract on revenue-sharing is always smaller than on wholesale price. This is mainly because in order to encourage the manufacturer to reduce carbon emissions, the retailer gives the manufacturer part of the revenue subsidies. If the cost of carbon emissions accounts for a high proportion of capital, the manufacturer may not participate in the production, resulting in the retailer having no goods to order and the retailer losing more. Secondly, with a contract on revenue-sharing, regardless of whether the manufacturer’s power control structure or the retailer power is dominant, with the increase of the revenue-sharing proportion, the profit of the retailer increases gradually. 

When the carbon emission level has a great impact on the demand, with a contract on revenue-sharing, regardless of whether the manufacturer or the retailer power is dominant, the profit of the retailer decreases first and then increases gradually with the increase of the revenue-sharing proportion. Therefore, the retailer can improve its profits by adjusting the proportion of revenue-sharing and can also encourage the manufacturer to reduce carbon emissions accordingly.

## 7. Conclusions

In the context of a low-carbon economy, this paper studies the green product pricing and carbon emission reduction of each member in a two-level supply chain composed of a single manufacturer and a single retailer under different power control structure modes. In the context of consumers with a preference for low-carbon products, firstly, the pricing and carbon emission reduction strategies of green supply chain members under different power control structures with a contract on wholesale prices are studied. Then, on this basis, a game model of carbon emission reduction with a contract on revenue-sharing is established, and the changes in the pricing and carbon emission reduction strategies of green supply chain members under different power control structures are studied. It is found that the wholesale price of the manufacturer with a contract on revenue-sharing is always higher than that with a contract on wholesale price, and it is inversely proportional to the revenue-sharing ratios, regardless of the manufacturer’s power control structure or the retailer power control structure. Under the two power control structures, the carbon emission level of the manufacturer with a contract on revenue-sharing is always lower than that with a contract on wholesale price, and it gradually decreases with the increase of the revenue-sharing proportion of the manufacturer. Once consumers’ awareness of low carbon is improved, it will improve the profits of the manufacturer. However, the impact of consumers low-carbon awareness on the profits of retailer depends on the proportion of revenue-sharing. Under different proportions of revenue-sharing, the impact of consumers’ low-carbon awareness on the profits of retailer is different. 

The results showed that when the retailer dominates the supply chain, the retailer selling price with a contract on revenue-sharing is always higher than on wholesale price. Under the manufacturer’s power control structure, when the revenue-sharing ratio is small, the retailer selling price with a contract on revenue-sharing is higher than on wholesale price; when the revenue-sharing ratio is large, the retailer selling price with a contract on revenue-sharing is lower than on wholesale price. Under the revenue sharing contract, the carbon emission level is increased, which has nothing to do with the power structure. When the supply chain adopts a revenue-sharing contract, although the profit of the retailer is reduced, the manufacturer will not be excluded. The retailer and the manufacturer should work together to improve the consumers’ low-carbon awareness and give the manufacturer income subsidies so that the manufacturer can make up for part of the carbon emission investment and then play an active role in a revenue-sharing contract. For example, Tsingtao Beer’s “low-carbon operation mode” not only carries out internal technology R&D (Research and Developmenand) product design, but also cooperates with back-end retailers to explore the development mode of a low-carbon economy. Retailers can promote low-carbon products by explaining and recommending low-carbon products to consumers, and effectively guide consumers’ green consumption concept.

The limitations of this paper are as follows: the supply chain members are assumed to be risk-neutral. There is no financial constraint on the manufacturer. Only wholesale price contracts and revenue risk contracts are considered. Future research can also consider the decision-making of supply chain members in relation to social preferences. In addition, a carbon emission cost-sharing contract can also be introduced to improve the operating environment of the green supply chain. The question of how to choose a revenue-sharing contract and cost-sharing contract will have a certain practical significance in future research.

## Figures and Tables

**Table 1:** The decisions of the supply chain under two different power structures with a wholesale price contract.

Structures or Variables	$w^{*}\;$	$e^{*}\;$	$p^{*}$
NM	$\frac{\gamma }{4k-\gamma^{2}}$	$\frac{2k}{4k-\gamma^{2}}$	$\frac{4k-\gamma^{2}+2k\gamma +\gamma }{2\left(4k-\gamma^{2}\right)}$
NR	$\frac{\gamma }{2\left(2k-\gamma^{2}\right)}$	$\frac{k}{2\left(2k-\gamma^{2}\right)}$	$\frac{\gamma +2k-\gamma^{2}}{2\left(2k-\gamma^{2}\right)}$

**Table 2:** The decisions of the supply chain under two different power structures with a revenue-sharing contract.

Structures or Variables	$w^{*}\;$	$e^{*}\;$	$p^{*}\;$
SM	$\frac{\gamma }{2k+2\theta k-\gamma^{2}}$	$\frac{2k\theta^{2}}{2k+2\theta k-\gamma^{2}}$	$\frac{2\gamma k\theta^{3}+2k\theta^{2}+\left(2k-\gamma^{2}\right)\theta +\gamma }{2\theta \left(2k+2\theta k-\gamma^{2}\right)}$
SR	$\frac{\left(2-\theta \right)\left(A_{1}\gamma^{4}+A_{2}\gamma^{3}+A_{3}\gamma^{2}+A_{4}\gamma \right)}{2\left(G_{1}\gamma^{3}+G_{2}\gamma^{2}+G_{3}\gamma +G_{4}\right)\left[\left(\theta^{2}-2\theta \right)\gamma^{2}+\left(1-\theta \right)\gamma +2k\theta \right]}$	$\frac{B_{1}\gamma^{4}+B_{2}\gamma^{3}+B_{3}\gamma^{2}+B_{4}\gamma -2k^{2}\theta }{2\left(G_{1}\gamma^{3}+G_{2}\gamma^{2}+G_{3}\gamma +G_{4}\right)\left[\left(\theta^{2}-2\theta \right)\gamma^{2}+\left(1-\theta \right)\gamma +2k\theta \right]}$	$\frac{\left(-2+\theta \right)\left(C_{1}\gamma^{4}+C_{2}\gamma^{3}+C_{3}\gamma^{2}+C_{4}\gamma +4k^{2}\theta \right)}{2\left(G_{1}\gamma^{3}+G_{2}\gamma^{2}+G_{3}\gamma +G_{4}\right)\left[\left(\theta^{2}-2\theta \right)\gamma^{2}+\left(1-\theta \right)\gamma +2k\theta \right]}$

**Table 3:** The influence of sharing ratios on the wholesale price under four modes (Unit: USD).

Structures or Variables		NM	SM	NR	SR
γ	θ	$w^{nm*}$	$w^{sm*}$	$w^{nr*}$	$w^{sr*}$
0.3	0.1	0.0767	0.1422	0.0785	0.1531
	0.3	0.0767	0.1195	0.0785	0.1701
	0.5	0.0767	0.1031	0.0785	0.1539
	0.7	0.0767	0.0906	0.0785	0.1270
	0.9	0.0767	0.0809	0.0785	0.0954
0.7	0.1	0.1994	0.4094	0.2318	0.5807
	0.3	0.1994	0.3318	0.2318	0.5552
	0.5	0.1994	0.2789	0.2318	0.4914
	0.7	0.1994	0.2405	0.2318	0.3992
	0.9	0.1994	0.2115	0.2318	0.2901

**Table 4:** The influence of sharing ratios on the emission-reduction level under four modes (Unit: m^3^).

Structures or Variables		NM	SM	NR	SR
γ	θ	$e^{nm*}$	$e^{sm*}$	$e^{nr*}$	$e^{sr*}$
0.3	0.1	0.5115	0.0095	0.2618	−0.1894
	0.3	0.5115	0.0717	0.2618	0.0646
	0.5	0.5115	0.1718	0.2618	0.1609
	0.7	0.5115	0.2961	0.2618	0.2097
	0.9	0.5115	0.4364	0.2618	0.2441
0.7	0.1	0.5698	0.0117	0.3311	−0.0433
	0.3	0.5698	0.0853	0.3311	0.1477
	0.5	0.5698	0.1992	0.3311	0.2640
	0.7	0.5698	0.3368	0.3311	0.3180
	0.9	0.5698	0.4894	0.3311	0.3318

**Table 5:** The influence of sharing ratios on the selling price under four modes (Unit: USD).

Structures or Variables		NM	SM	NR	SR
γ	θ	$p^{nm*}$	$p^{sm*}$	$p^{nr*}$	$p^{sr*}$
0.3	0.1	0.6151	1.2123	0.5785	1.1198
	0.3	0.6151	0.7100	0.5785	0.8231
	0.5	0.6151	0.6289	0.5785	0.6972
	0.7	0.6151	0.6091	0.5785	0.6297
	0.9	0.6151	0.6104	0.5785	0.5907
0.7	0.1	0.7991	2.5509	0.7318	1.5939
	0.3	0.7991	1.0828	0.7318	1.3296
	0.5	0.7991	0.8486	0.7318	1.1034
	0.7	0.7991	0.7897	0.7318	0.9219
	0.9	0.7991	0.7888	0.7318	0.7846

**Table 6:** The influence of sharing ratios on the profit of the manufacturer under four modes (Unit: USD).

Structures or Variables		NM	SM	NR	SR
γ	θ	$M^{nm*}$	$M^{sm*}$	$M^{nr*}$	$M^{sr*}$
0.3	0.1	−0.0895	−0.2584	0.0050	−0.2230
	0.3	−0.0895	0.1895	0.0050	0.1444
	0.5	−0.0895	0.1617	0.0050	0.1635
	0.7	−0.0895	0.0873	0.0050	0.1149
	0.9	−0.0895	−0.0215	0.0050	0.0447
0.7	0.1	−0.0427	−4.1733	0.0611	−4.1733
	0.3	−0.0427	−0.0288	0.0611	−0.0288
	0.5	−0.0427	0.1847	0.0611	0.1847
	0.7	−0.0427	0.1563	0.0611	0.1563
	0.9	−0.0427	0.0410	0.0611	0.0410

**Table 7:** The influence of sharing ratios on the profit of the retailer under four modes(Unit: USD).

Structures or Variables		NM	SM	NR	SR
γ	θ	$R^{nm*}$	$R^{sm*}$	$R^{nr*}$	$R^{sr*}$
0.3	0.1	0.2898	0.0044	0.2500	0.0073
	0.3	0.2898	0.0291	0.2500	0.0151
	0.5	0.2898	0.0893	0.2500	0.0684
	0.7	0.2898	0.1611	0.2500	0.1359
	0.9	0.2898	0.2439	0.2500	0.2105
0.7	0.1	0.3597	0.2380	0.2500	0.2630
	0.3	0.3597	0.0016	0.2500	0.0354
	0.5	0.3597	0.0423	0.2500	0.0049
	0.7	0.3597	0.1393	0.2500	0.0740
	0.9	0.3597	0.2760	0.2500	0.1862
